# Differentiation Between Disseminated Carcinomatosis of the Bone Marrow From Urothelial Cancer and Intravascular Large B-cell Lymphoma: A Case Report

**DOI:** 10.7759/cureus.57221

**Published:** 2024-03-29

**Authors:** Ryuichi Ohta, Nozomi Nishikura, Shinichiro Suyama, Chiaki Sano

**Affiliations:** 1 Community Care, Unnan City Hospital, Unnan, JPN; 2 Internal Medicine, Heisei Memorial Hospital, Unnan, JPN; 3 Community Medicine Management, Shimane University Faculty of Medicine, Izumo, JPN

**Keywords:** intravascular large b-cell lymphoma, general medicine, rural, elderly patient care, diagnostic challenges, tafro syndrome, urothelial carcinoma, bone marrow neoplasms, disseminated carcinomatosis

## Abstract

This case report describes a rare case of intravascular large B-cell lymphoma (IVLBCL), initially presenting with nonspecific symptoms of fever and fatigue, and tentatively diagnosed as disseminated carcinomatosis of the bone marrow originating from urothelial cancer in an 80-year-old woman. The patient’s journey began with symptoms treated as common ailments and progressed through multiple differential diagnoses, including giant cell arteritis, TAFRO (thrombocytopenia, anasarca, fever, reticulin fibrosis, and organomegaly) syndrome, and disseminated carcinomatosis of the bone marrow originating from urothelial cancer due to the presence of systemic inflammation, anasarca, and elevated soluble interleukin 2 receptor levels, indicative of an intense immunological response. Despite initial treatments, her condition deteriorated, leading to further investigations that ultimately revealed the presence of malignant cells in the urine and bone marrow, confirming the diagnosis of IVLBCL. This case underscores the diagnostic challenges faced when elderly patients present with systemic inflammation and the critical need for thorough investigation beyond initial impressions. It highlights the importance of considering differentiation between disseminated carcinomatosis of the bone marrow and IVLBCL in the differential diagnosis of persistent inflammation, especially in cases where common causes have been excluded and the primary malignancy is not immediately apparent.

## Introduction

Disseminated carcinomatosis of the bone marrow represents an uncommon clinical manifestation of cancer, predominantly observed in the context of advanced disease stages where cancer has metastasized, typically sparing the bone [1,2]. The frequency is 0% to 2% among solid tumors [1,2]. This condition is characterized by the widespread dissemination of cancer cells within the bone marrow, affecting the normal hematopoiesis process and leading to a variety of systemic symptoms [3]. While most cases of disseminated carcinomatosis of the bone marrow are associated with primary malignancies of the prostate, lung, breast, and stomach, instances stemming from urothelial cancer, mainly originating from sigmoid colon cancer, are notably rare [4,5]. The differentiation from intravascular lymphoma, the frequency of which is also rare with less than one in one million general population, is challenging, especially when patients’ conditions are critical and adequate investigations are impossible [6].

In the present case report, we delineate an exceptional occurrence of intravascular B-cell lymphoma (IVLBCL) demanding the differentiation from disseminated carcinomatosis of the bone marrow from urothelial cancer. The patient, presenting with symptoms of fever and fatigue, was found to have an extraordinarily elevated level of soluble interleukin 2 receptor, a marker indicative of an intense immunological response [6]. This finding underscores the significance of considering disseminated carcinomatosis of the bone marrow and IVLBCL as a potential diagnosis in older patients exhibiting similar clinical presentations, even when the primary cancer is less commonly associated with this condition.

## Case presentation

An 80-year-old woman with full activity in daily life and living by herself came to a rural community hospital with a chief complaint of fatigue and systemic pain for two months. Two months before admission, the patient had a low-grade fever and fatigue and was treated with acetaminophen 500 mg by her primary care physician for a month. However, her symptoms were gradually progressive. One month before admission, she visited a rural hospital for further investigation. As chest computed tomography (CT) was performed to clarify infiltrations around bronchioles on bilateral lungs, she was diagnosed with bronchial pneumonia and treated with intravenous ceftriaxone 2 g and levofloxacin 500 mg daily for one week. However, her fever was persistent at around 38°C. As she began to have polyarthritis and muscular pains, she was suspected of pseudogout and polymyalgia rheumatica and treated with prednisolone 20 mg daily for one week. The symptoms of fever and fatigue improved, but with the cessation of prednisolone, the symptoms reemerged. The laboratory data showed thrombocytopenia and anemia. Additional tests for the investigation showed an extremely high level of soluble interleukin 2 receptor of 23,010 U/mL. Suspecting the possibility of intravascular lymphoma and other autoimmune diseases, she was transferred to a rural general hospital. The past medical histories were hypertension and dyslipidemia. She was treated with enalapril 5 mg daily and atorvastatin 5 mg daily, respectively.

The vital signs at the visit were as follows: blood pressure, 90/60 mmHg; pulse rate, 105 beats/minute; body temperature, 37.2°C; respiratory rate, 24 breaths/minute; and oxygen saturation, 97% on room air. The patient was alert to time, place, and person. Physical examination showed systemic edema and polyarticular tenderness of bilateral shoulders, elbows, knees, and ankles. No other abnormal neurological findings were noted. There were no obvious abnormalities in the chest, abdomen, or skin. The laboratory tests showed high inflammatory conditions with thrombocytopenia, anemia, and hypogammaglobinemia (Table 1).

**Table 1:** Initial laboratory data of the patient. PT = prothrombin time; INR = international normalized ratio; APTT = activated partial thromboplastin time; eGFR = estimated glomerular filtration rate; CK = creatine kinase; CRP = C-reactive protein; TSH = thyroid-stimulating hormone; Ig = immunoglobulin; HCV = hepatitis C virus; SARS-CoV-2 = severe acute respiratory syndrome coronavirus 2; HBs = hepatitis B surface antigen; HBc = hepatitis B core antigen; C3 = complement component 3; C4 = complement component 4; MPO-ANCA = myeloperoxidase antineutrophil cytoplasmic antibody; CCP = cyclic citrullinated peptide

Parameter	Level	Reference
White blood cells	15.20	3.5–9.1 × 10^3^/μL
Neutrophils	88.7	44.0–72.0%
Lymphocytes	3.6	18.0–59.0%
Monocytes	7.3	0.0–12.0%
Eosinophils	0.1	0.0–10.0%
Basophils	0.3	0.0–3.0%
Red blood cells	2.48	3.76–5.50 × 10^6^/μL
Hemoglobin	8.1	11.3–15.2 g/dL
Hematocrit	24.5	33.4–44.9%
Mean corpuscular volume	98.9	79.0–100.0 fL
Platelets	4.7	13.0–36.9 × 10^4^/μL
Erythrocyte sedimentation rate	31	2–10 mm/hour
Total protein	4.2	6.5–8.3 g/dL
Albumin	2.7	3.8–5.3 g/dL
Total bilirubin	2.2	0.2–1.2 mg/dL
Direct bilirubin	0.8	0.0–0.4 mg/dL
Aspartate aminotransferase	11	8–38 IU/L
Alanine aminotransferase	12	4–43 IU/L
Alkaline phosphatase	58	106–322 U/L
Lactate dehydrogenase	209	121–245 U/L
Blood urea nitrogen	33.3	8–20 mg/dL
Creatinine	0.91	0.40–1.10 mg/dL
eGFR	45.1	>60.0 mL/min/L
Serum Na	134	135–150 mEq/L
Serum K	4.3	3.5–5.3 mEq/L
Serum Cl	92	98–110 mEq/L
Serum Ca	8.4	8.8–10.2 mg/dL
Serum P	3.6	2.7–4.6 mg/dL
Serum Mg	1.9	1.8–2.3 mg/dL
Ferritin	1,006.4	14.4–303.7 ng/mL
CK	18	56–244 U/L
CRP	2.39	<0.30 mg/dL
TSH	1.12	0.35–4.94 μIU/mL
Free T4	1.1	0.70–1.48 ng/dL
IgG	614	870–1,700 mg/dL
IgM	31	35–220 mg/dL
IgA	108	110–410 mg/dL
IgE	13	<173 mg/dL
HBs antigen	0.0	IU/mL
HBs antibody	0.00	mIU/mL
HBc antibody	0.00	S/CO
HCV antibody	0.00	S/CO
Syphilis treponema antibody	0.00	S/CO
SARS-CoV-2 antigen	-	
Anti-nuclear antibody	40	<40
C3	45	86–164 mg/dL
C4	10	17–45 mg/dL
MPO-ANCA	<1.0	<3.5 U/mL
Anti-CCP antibody	<0.6	<5 U/mL
Anti-cardiolipin antibody IgG	8.2	<12.3 U/mL
Soluble interleukin receptor antibody	24,722	122–496 U/mL
Urine test		
Leukocyte	Negative	Negative
Nitrite	Negative	Negative
Protein	Negative	Negative
Glucose	Negative	Negative
Urobilinogen	Normal	
Bilirubin	Negative	Negative
Ketone	Negative	Negative
Blood	Negative	Negative
pH	6.5	
Specific gravity	1.013	

**Figure 1:**
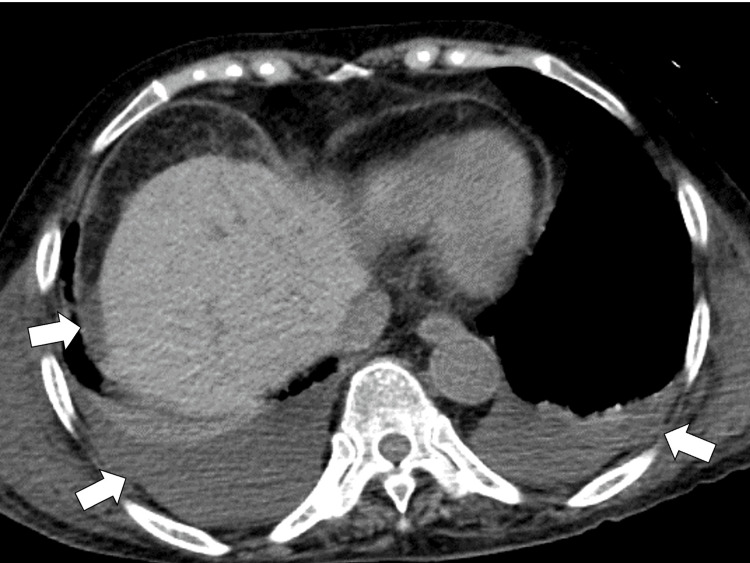
Neck-to-pelvic computed tomography clarifying bilateral pleural effusion and ascites (white arrows).

Neck-to-pelvic CT was performed to investigate the etiology of systemic inflammation, clarifying bilateral pleural effusion, ascites, and mild hepatosplenomegaly without any lymphadenopathy (Figure 1).

From vital signs and immunocompromised condition, we suspected sepsis from bacterial translocation. The therapy was initiated with meropenem 3 g daily for one week and stopped after the reports of the negative results of blood and urine cultures. On the same day, a random skin biopsy and bone marrow test were performed, clarifying that there were no typical findings of intravascular lymphoma. Because of the shock status, the bone marrow biopsy could not be performed.

On day three of the admission, systemic muscular pain, and headache were progressive, and a temporal artery ultrasound showed edematous changes in the perivascular regions of the bilateral temporal arteries. We suspected giant cell arteritis (GCA) and increased the dose of prednisolone to 50 mg daily.

On day eight of the admission, after the usage of the antibiotic and the increase in the dosage of prednisolone, her appetite continually decreased, and systemic edema exacerbated. The neck-to-pelvic CT scan was performed, clarifying the increase in pleural effusion and ascites. We suspected TAFRO (thrombocytopenia, anasarca, fever, reticulin fibrosis, and organomegaly) syndrome based on the diagnostic criteria of fever, renal damage, pleural effusion, ascites, mild hepatosplenomegaly, and thrombocytopenia [7]. Through the discussion with the patient and families, we decided to use tocilizumab 400 mg intravenously.

**Figure 2:**
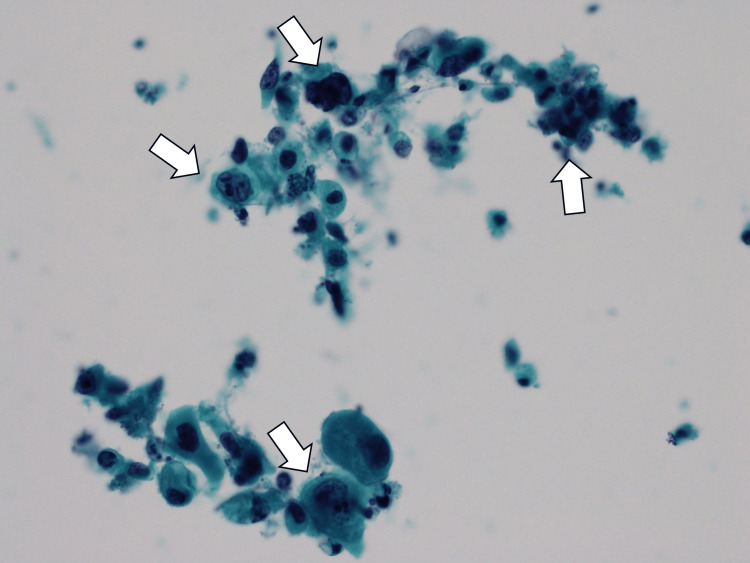
Urinalysis clarifying massive malignant urethral cells (white arrows).

On day nine of the admission, her urinalysis clarified massive malignant urethral cells (more than 30 cells/high-power field) (Figure 2).

**Figure 3:**
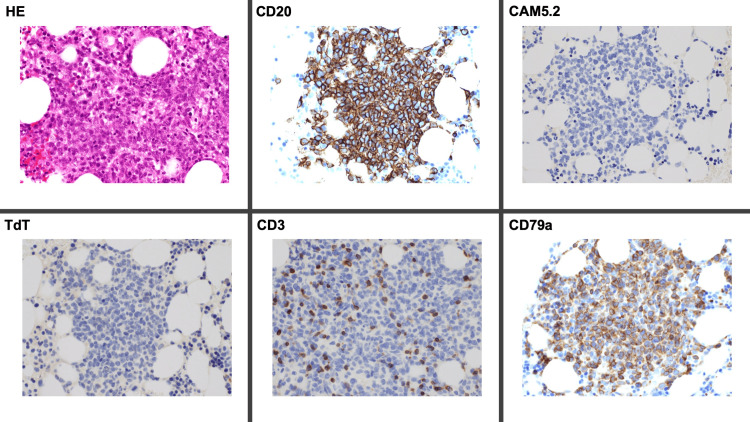
Immunological stains of the bone marrow showing the increase in cells predominantly positive for B-cell markers of CD20 and CD79a without the positivity of CD3, terminal deoxynucleotidyl transferase, and keratin CAM5.2. CAM5.2 = keratin CAM5.2; CD = classification of differentiation; HE = hematoxylin and rosin; TdT = terminal deoxynucleotidyl transferase

The repeat CT showed no abnormality of the kidneys, urethra, bladder, and ureter without any lymphadenopathy. Eventually, she was diagnosed tentatively with disseminated carcinomatosis of the bone marrow from urothelial cancer. On day 11 of admission, she developed disseminated intravascular coagulation. The patient and her family chose palliative care. On day 14 of the admission, the patient died. After the patient’s death, the result of immunological stains of the previous bone marrow was clarified (Figure 3).

The immunological stain showed an increase in cells predominantly positive for B-cell markers of classification of differentiation (CD)20 and CD79a without the positivity of CD3, terminal deoxynucleotidyl transferase, and keratin CAM5.2, suspecting IVLBCL.

## Discussion

In this case report, we delve into the complexities and diagnostic challenges encountered when dealing with inflammation of unknown origins in an elderly patient, ultimately revealing a rare manifestation of IVLBCL accompanied by urothelial cancer. The patient’s initial presentation and the subsequent clinical course underscore the diagnostic conundrum clinicians face, initially leading to tentative diagnoses of GCA, TAFRO syndrome, and disseminated carcinomatosis of the bone marrow before arriving at the definitive diagnosis. This journey highlights the necessity of maintaining a broad differential diagnosis and the importance of comprehensive diagnostic evaluations in such complex cases.

Expanding on the initial consideration, it is essential to note that GCA is a vasculitis of large and medium-sized vessels, most notably affecting the temporal arteries, and is characterized by systemic inflammation and elevated inflammatory markers such as erythrocyte sedimentation rate and C-reactive protein [8,9]. The American College of Rheumatology criteria for classifying GCA emphasize the role of clinical symptoms, elevated erythrocyte sedimentation rate, and abnormal artery biopsy findings in diagnosis [10]. The standard treatment involves high-dose corticosteroids, typically prednisolone, which are effective in many cases [11,12]. However, as our case shows, a subset of patients may not respond adequately or may experience relapse upon tapering of steroids, highlighting the limitations of clinical and laboratory findings in distinguishing GCA from other causes of systemic inflammation.

TAFRO syndrome, characterized by thrombocytopenia, anasarca, fever, renal dysfunction, and organomegaly, presents a diagnostic challenge due to its nonspecific criteria and recent description in the medical literature [13,14]. Our case fits the diagnostic criteria. The previous articles described TAFRO syndrome as part of the spectrum of multicentric Castleman’s disease but distinct in its clinical presentation and management challenges [13-15]. This underscores the necessity for cautious application of diagnostic criteria and the importance of comprehensive evaluation and discussion with patients and their families regarding the treatment options, given the syndrome’s potential overlap with other conditions.

The diagnosis of disseminated carcinomatosis of the bone marrow from urothelial cancer in our patient underscores the diverse presentation of cancer, particularly the rare occurrence of cancer cells disseminating to the bone marrow from a primary urothelial carcinoma [16]. Soluble interleukin 2 receptor can increase highly in some cases in aggressive urothelial cancers [6,16]. Such cases highlight the need for high clinical suspicion and systematic investigation in patients presenting with unexplained systemic inflammation in primary care settings and general medicine [17]. A previous study on the rarity of bone marrow metastases in urothelial carcinoma emphasizes the importance of considering metastatic disease in the differential diagnosis of systemic symptoms, particularly in older patients or those with atypical presentations, underscoring the need for a comprehensive approach to diagnosis and management [16]. Cancers can develop anywhere in human bodies and metastases in any way, so general physicians should systematically investigate human bodies with inflammation of unknown origins even if there are no suspected regions in imaging tests [17].

Finally, IVLBCL is a rare extranodal diffuse large B-cell lymphoma, primarily affecting the small vessels. It often presents with nonspecific symptoms, making diagnosis challenging [18,19]. The role of bone marrow and skin biopsies in IVLBCL diagnosis is critical, as highlighted by previous articles, which emphasized that IVLBCL can present with various nonspecific symptoms, including fever of unknown origin. That biopsy of involved tissues often provides a definitive diagnosis [20]. However, in our case, the biopsy of the bone marrow could be performed because of the critical condition of the patient. In our case, the initial negative result of the bone marrow could not rule out the likelihood of IVLBCL, underscoring the importance of these diagnostic procedures in excluding IVLBCL among the differential diagnoses.

## Conclusions

This case report sheds light on a rare clinical presentation of IVLBCL and emphasizes the intricate process involved in diagnosing conditions with nonspecific symptoms. Older patients can have multiple diseases simultaneously. This case is a valuable learning point for general physicians and specialists, highlighting the complexities of diagnosing and managing inflammation of unknown origins, the pivotal role of clinical vigilance and comprehensive evaluation, and the limitations of geriatric care.
